# Morphogenesis, Growth Cycle and Molecular Regulation of Hair Follicles

**DOI:** 10.3389/fcell.2022.899095

**Published:** 2022-05-12

**Authors:** Xiangyu Lin, Liang Zhu, Jing He

**Affiliations:** Department of Pathology and Pathophysiology, Shanghai Skin Disease Hospital, School of Medicine, Tongji University, Shanghai, China

**Keywords:** hair follicles, morphogenesis, hair cycling, WNT, BMP

## Abstract

As one of the main appendages of skin, hair follicles play an important role in the process of skin regeneration. Hair follicle is a tiny organ formed by the interaction between epidermis and dermis, which has complex and fine structure and periodic growth characteristics. The hair growth cycle is divided into three continuous stages, growth (anagen), apoptosis-driven regression (catagen) and relative quiescence (telogen). And The Morphogenesis and cycle of hair follicles are regulated by a variety of signal pathways. When the signal molecules in the pathways are abnormal, it will affect the development and cycle of hair follicles, which will lead to hair follicle-related diseases.This article will review the structure, development, cycle and molecular regulation of hair follicles, in order to provide new ideas for solving diseases and forming functional hair follicle.

## 1 Introduction

As the first barrier against external environmental damage, the skin is composed of three layers. The first is the outermost epidermis, consisting of cycling keratinocytes that pile up and transitions into outer layers of dead, cornified keratinocytes that provide the protection against environmental insult and loss of moisture. The second layer underlying the epidermis is the dermis, which contains the skin appendages, including hair follicles, sebaceous glands (SGs), eccrine glands, and apocrine sweat glands. Nails, which are appendages found at the ends of digits, also arise from the dermis. Finally, the deepest layer of the skin underlying the dermis is the subcutaneous tissue, which consists of insulating adipose tissue and connective tissue that connects the skin to the tissue underneath the skin. Blood vessels and nerves in the subcutaneous tissue provide the source of capillaries and nerve endings that penetrate into the dermis and interact with the appendages (Stephens, 2022).

Hair follicles, as one of the important skin appendages, plays an irreplaceable role in skin function and in the process of skin regeneration. The hair follicle is a unique skin structure found in mammals, and is essentially a small organ formed by the interaction between epidermis and dermis. Hair follicles contain many components and have complex, fine structures. They have a high capacity of self-renewal, and display a periodic growth cycle that takes place continually throughout the life span of mammalian organisms. The hair follicle is rich in stem cell populations that contribute not only to hair growth and regeneration but also contribute to skin regeneration after injury. Thus, hair follicles can serve as important models for tissue regeneration and systems biology research (Ma et al., 2017). The growth of hair follicles and activity of these stem cells is highly regulated by various signaling pathways. Hair growth is affected by many factors such as age, climate, environment, and health status, and these factors can influence the development of hair follicle tumors, alopecia areata, and other related diseases.

## 2 Structure of Hair Follicles

**FIGURE 1:**
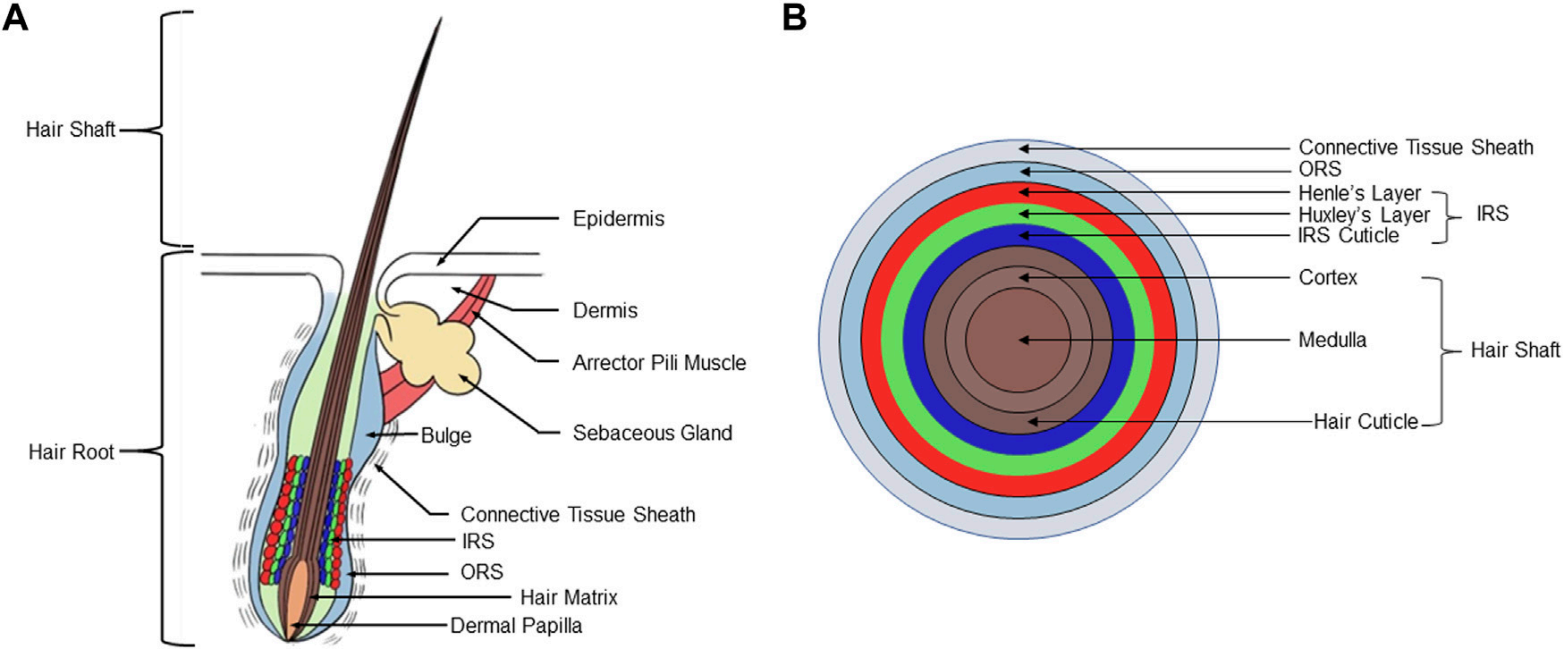
Structure of hair follicle. **(A)** A human scalp hair follicle (anagen VI): the permanent (infundibulum, isthmus) and anagen associated (suprabulbar and bulbar area) components of the hair follicle. **(B)** Schematic diagram of the concentric layers of the hair follicle bulb, including hair shaft, IRS, outer root sheath, and connective tissue sheath. IRS, inner root sheath.

As the largest organ of the human body, the skin is mainly composed of epidermis and dermis (Souto et al., 2022) (Figure 1A). The epidermis can be further divided into sublayers consisting of, from external to basal, the stratum corneum, stratum lucidum, stratum granulosum, stratum spinosum, and the stratum basale. The dermis is located immediately beneath the stratum basale and consists of papillary layer and reticular layer. Subcutaneous tissue beneath the dermis, also termed the hypodermis, mainly includes loose connective tissue and adipose tissue. Although the shape and size of hair follicles may vary considerably depending on their specific location in the body, they all have the same basic structure (Morita et al., 2021) (Figure 1B). The hair follicles run obliquely in the skin. On the obtuse side of the skin surface, there is a bundle of smooth muscle connecting the hair follicle and the papillary layer of the dermis, called the arrector pili muscle (APM). APM is innervated by the sympathetic nervous system. When the APM contracts, it erects the hair and promotes secretion from associated sebaceous gland (SG). The hair follicle is divided into four regions from top to bottom: infundibulum, isthmus, suprabulbar region, and the bulb. The region from the opening of the hair follicle to the opening of the SG is called the infundibulum, the region from the opening of the SG to the attachment of the APM is called the isthmus. Beneath the isthmus, starting at the attachment site of the APM, is the suprabulbar region, which terminates in an enlarged, spherical structure called the bulb (Carrasco et al., 2019).

The composition of cells in the upper half of the hair follicle, the infundibulum, and isthmus, is relatively constant. However, the isthmus does contain a population of stem cells that can help to re-populate the epidermis during wound healing. Studies have identified a population of Gli1^+^Lgr6^+^ cells in the isthmus which can contribute to formation of wound epithelium, and can provide a source of long-lasting epithelial precursors in healed epidermis (Snippert et al., 2010; Huang S. et al., 2021). The bulge region is located at the junction of the APM and the ORS. Cotsarelis et al. (1990) based on experiments labeling skin hair follicle cells with 3H-TdR, were the first to propose that hair follicle stem cells (HFSCs) are contained in the hair follicle bulge region. HFSCs have typical stem cell characteristics, are highly proliferative, and are critical for the maintenance of hair growth and renewal. Studies by Hsu et al. (2011) show that the periodic growth of hair follicles depends on the maintenance of HFSCs, which can participate in the formation of hair follicles, the maintenance of SGs, and the renewal of the epidermis. For example, krt15^+^ HFSCs of the bulge can provide progeny to help rapidly populate the wound epithelium and repair the epidermis (Ito et al., 2005; Yu et al., 2020). Festa et al. (2011) have found that the formation of fat *in vivo* is synchronized with the activation of HFSCs, and the number of subcutaneous adipose precursor cells reaches a peak during the activation of HFSCs.

Composition of the lower region of the hair follicle is much more variable, including differentiated epithelial cells, hair matrix, and dermal papilla (DP) (O'Sullivan et al., 2021). The bulb is located at the lowest end of the hair follicle and is the active growth center of the hair. Hair follicles are obliquely rooted in the dermis, and the dermis plays a key role in supplying nutrients for hair follicle growth and development (Lee et al., 2020). The upwardly-directed indentation in the bottom of the bulb is the DP, formed by an intrusion of connective tissue, which contains a rich supply of capillaries and nerve endings (Park et al., 2018). The DP supplies nutrition for hair growth and maintenance of the follicle. The DP is a multicellular tissue structure formed by the aggregation of dermal cells, which plays an important role in inducing hair growth (Ge et al., 2020). Dermal stem cells are a kind of skin stem cells with self-renewal ability, dermal cup and lower dermal sheath harbors dermal stem cells which regenerate a new dermal sheath and repopulates cells into the DP during hair cycling (Rahmani et al., 2014). Injury of hair follicles was shown to recruit more dermal stem cell progeny to become DP cells (Sparks et al., 2019). Matsuzaki et al. showed that mouse dermal papilla cells (DPCs) cultured *in vitro* still retain the ability to induce hair follicle formation *in vivo*, which provides an experimental basis for hair follicle reconstruction (Matsuzaki and Yoshizato, 1998). Oliver’s study found that after removing the lower third of the hair follicles of mouse vibrissa, the DP will regenerate and produce vibrissa, but if more of the hair follicles are removed, vibrissa will not regenerate (Oliver, 1966). If more than one-third of the lower part of the hair root is removed from the hair follicles, the vibrissa can be regenerated after the hair papilla is implanted into the base of the hair follicles (Oliver, 1967).

The hair matrix cells are located in the upper part and lateral side of the DP, and melanocytes are scattered between them. DPCs can induce the formation of epithelial components such as inner root sheath (IRS) and medulla of the shaft by interacting with their surrounding hair matrix cells (Limbu and Higgins, 2020). From the upper section of the bulb to the upper part of the hair follicle, it presents a concentric circle-shaped layer, which is divided into three parts from inside to outside: hair shaft (HS), IRS, outer root sheath (ORS). The HS is the part exposed to the skin and is composed of keratinocytes. From inside to outside, the layers of the shaft are the medulla, cortex, and hair cuticle (Watanabe et al., 2021). The IRS consists of the IRS cuticle, Huxley’s layer, Henle’s layer, and companion layer from inside to outside (Watanabe et al., 2021). The ORS is produced by the Malpighian layer of epidermis (Nilforoushzadeh et al., 2020). The IRS and the ORS are collectively called the epithelial sheath, which belongs to the epidermal component of the hair follicle. The outermost layer of the hair follicle is called the connective tissue sheath, and is also known as the dermal sheath. This layer is derived from the mesenchymal hair follicle dermis component, and is composed of three layers of collagen fibers arrayed in different directions (Martino et al., 2021). The connective tissue sheath is an important material basis for maintaining and regenerating dermal papillae, and is a necessary structure for hair follicle regeneration (Heitman et al., 2020). SGs are located in the dermis of the skin, and their ducts open between the isthmus and infundibulum of the hair follicle (Geueke and Niemann, 2021).

## 3 Morphogenesis of Hair Follicles

Hair follicles are micro organs-formed by the interaction between epidermis and dermis (Ji et al., 2021). Thus, these structures are composed of cells that arise from two different embryonic tissue sources, ectoderm and mesoderm (Ji et al., 2021). Epidermal stem cells and neural crest stem cells are derived from ectoderm (Yang et al., 2019; Soto et al., 2021), while mesenchymal stem cells arise from mesoderm (Schaefer et al., 2020). Epidermal-derived cell lines include SG cells and keratinocytes. Keratinocytes can further differentiate into IRS cells, ORS cells, and hair cells (Morgan et al., 2020). Mesenchymal-derived cell lines include fibroblasts and connective tissue sheath cells (Wang et al., 2018; Hernaez-Estrada et al., 2022). Neural crest cells can form melanocytes, which are the source of hair pigment (Gacem et al., 2020).

**FIGURE 2:**
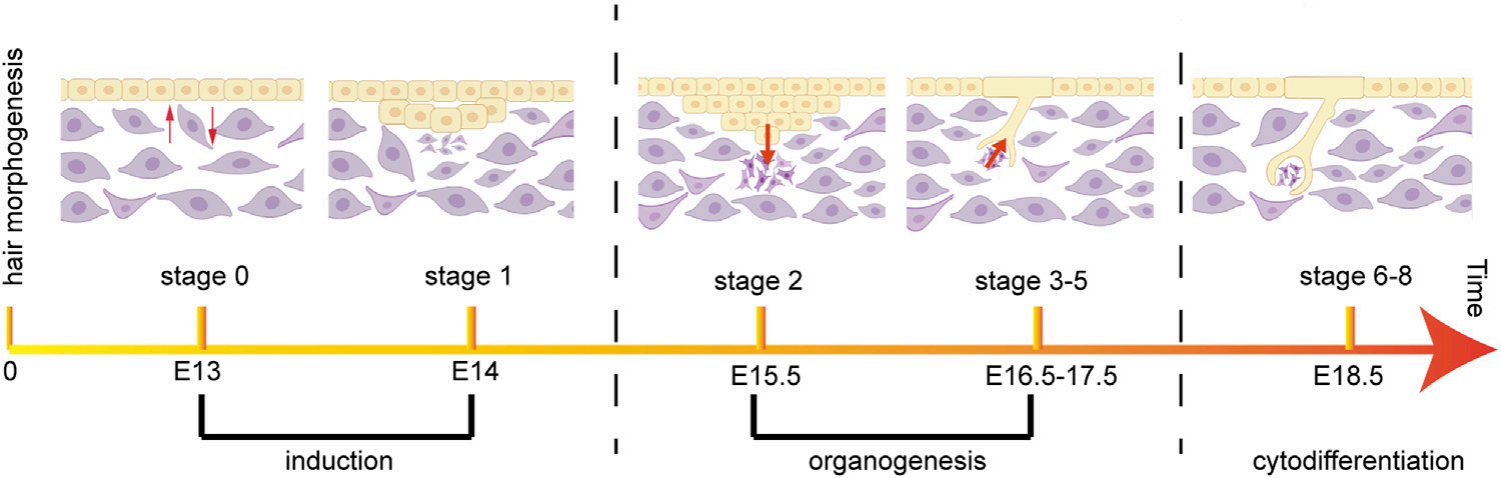
Morphogenesis and timing of hair follicle during mouse embryonic development. The most important developmental stages of mouse pelage hair follicles are divided into induction, organogenesis and cytodifferentiation.

The development of hair follicles is essentially a three-step process: induction, organogenesis, and cytodifferentiation, and these three steps include eight stages (Carbonnel et al., 2020; Schmidt-Ullrich and Paus, 2005) (Figure 2). During embryonic development, the morphogenesis of hair follicles depends on the regulation of a series of signals between dermis and epidermis. They mediate the interaction between dermal and epidermal cells, induce the orderly proliferation and differentiation of the two cell populations, and guide the cells to finally form HS, root sheath, and DP (Mapar et al., 2021). In the first stage, when signals inducing hair follicle cell generation are emitted from dermal cells, epithelial cells receiving dermal cell signals gradually thicken and form hair follicle basal plates (Zhao et al., 2021). The hair follicle basal plates will send out relevant signals to induce a large number of dermal cells to aggregate under the hair follicle basal plates, and a brand-new hair follicle will be generated at this aggregation point. In the second stage, when a critical density of dermal cells converge under the hair follicle basal plates, the dermal cells will send a signal to induce the hair follicle basal plate to expand downwards, so that the hair follicle structure can enter the dermis and form hair buds. Hair buds deep in the dermis gradually become columnar structures, and a large number of dermal fibroblast cells accumulate at the end (Paus et al., 1999). After entering the third stage, the hair buds are deeply sunk in the dermis layer, and keratinocytes are arranged in a columnar shape around the hair buds. In the fourth stage, the hair buds continue to thicken, and dermal cells converge under the basal plate of hair follicles to form dermal papillae. The formation of a hair bulge occurs in the fifth stage. At the same time, the DP induces the proliferation of hair matrix cells, which further differentiate into HS and IRS (Houschyar et al., 2020). In the sixth stage, hair follicle accessory organs and complete hair follicle structures differentiated from epithelial cells have been formed. In the seventh stage, the tip of the hair enters the hair canal, and the SGs are fixed on the wall of the hair follicle. In the eighth stage, the hair follicles have completed their development, and the HSs pass through the surface of the epidermis (Lee et al., 2018).

During the morphogenesis of mouse hair follicles, induction includes the first stage, which occurs on the 14th day of embryonic development. Organogenesis includes the second to the fifth stage, the second stage occurs at day 15.5, and the third to fifth stage occurs between days 16.5–17.5. Finally, cytodifferentiation takes place from stage 6 to stage 8, which occurs at 18.5 days of embryonic development (Schmidt-Ullrich and Paus, 2005) (Figure 2).

## 4 Hair Follicle Cycling

**FIGURE 3:**
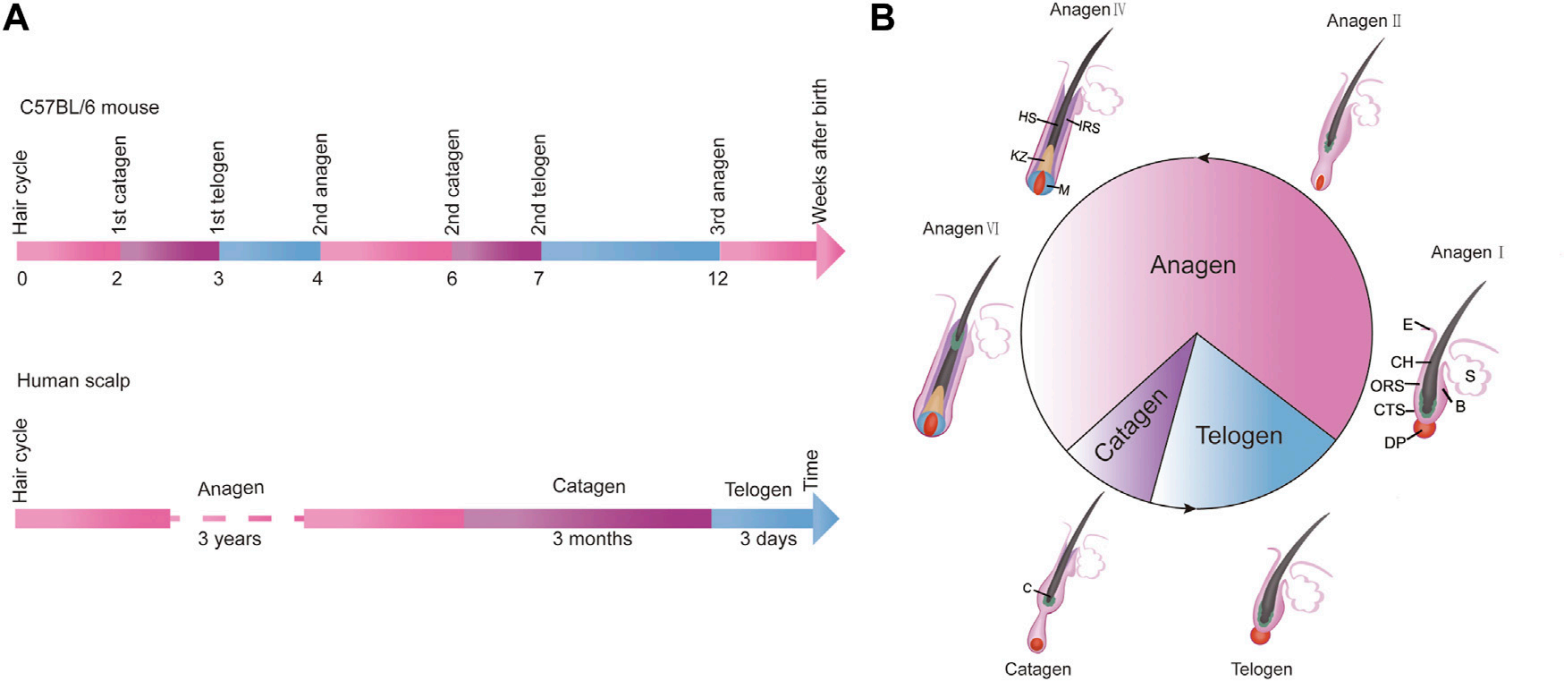
The growth cycle of hair. **(A)** The time-scale for the hair cycle in female C57BL/6 mice during the first 14 weeks after birth (the upper part); The time-scale for the hair cycle in humans (the lower part). **(B)** The morphology of hair follicles at different stages of the hair cycle. Anagen: growth phase, catagen: regression phase, telogen: resting phase.

Hair follicles go through regular growth cycles throughout the whole life process, and the changes that occur during the cycle are mainly changes in the morphology and structure of dermal papillae at the bottom of hair follicles, the formation of new HS, and the shedding of old hair. This cycle is divided into three stages: anagen, catagen, and telogen (Shin et al., 2020). Under typical conditions, the time scale of each cycle is relatively constant and precise. For example, C57BL/6 mice have a precise time scale for the occurrence of anagen, catagen, and telogen of hair follicles. Newborn mice enter catagen in the second week after birth, telogen in the third week, and the anagen in the fourth week (Chen et al., 2019) (Figure 3A). On the scalp of an adult, anagen lasts for approximately 3 years, followed by a catagen of about 3 weeks, and then a telogen of about 3 months (Grymowicz et al., 2020; Oh et al., 2016) (Figure 3B). Of course, the progress and timing of the three stages of the hair follicle growth cycle can also be affected by many factors: genetic background, environmental factors, gender factors, nutritional factors, and others (Muller-Rover et al., 2001). The hair growth cycle of different strains of mice was different, and the skin color of C57BL/6 mice changed with different hair follicle growth periods. Temperature and light can also affect the growth of hair follicles. Studies have shown that red light at 650 nm can promote the proliferation of human hair follicle cells and significantly delay the transition of hair follicles from anagen to telogen (Yang et al., 2021). The influence of gender factors on hair growth cycle is mainly regulated by hormones, and androgen has a high influence on hair growth and cycle (Grymowicz et al., 2020). At the same time, the regular growth cycle of hair can not be separated from the nutrition supply and regulation of peripheral nerves of hair follicles (Zhang J. et al., 2021).

### 4.1 Anagen

The anagen stage is the most active period of hair follicle growth, at which time the hair grows rapidly and forms a complete HS (Suen et al., 2020). The proliferation of secondary hair bud cells near the DP marks the beginning of anagen, and the hair follicles penetrate into the subcutaneous tissue. The bulb cells proliferate rapidly, the HS and IRS cells begin to differentiate, and the morphology and volume of DPCs and bulbs become larger (Vishlaghi and Lisse, 2020). Histologically, hair follicles during anagen are slender and straight, and the follicles are oriented at an angle so that the hair can be laid flat on the surface of the body. The keratinocyte progenitor cells in the matrix migrate to the top of the hair follicle and differentiate into HS and IRS cells. When HS cells enter terminal differentiation, they will bind closely with cysteine-rich hair keratin to form 10 nm bundle-like filaments. This cross-linking gives the HS a characteristic tensile strength and flexibility. The IRS can also be keratinized, which can support and guide the growth of HS in the process of HS differentiation. During anagen, the cell cycle of highly proliferative stromal cells is about 18 h (Harland, 2018). The time of growth cycle determines the length of hair and is related to the continuous proliferation and differentiation of stromal cells at the base of hair follicles (Morgun and Vorotelyak, 2020).

### 4.2 Catagen

The typical characteristics of hair follicles entering catagen are that HS stops growing, cell proliferation and differentiation ability begins to decline, cells begin to undergo apoptosis, and hair follicles rapidly degenerate. Apoptosis occurs in the epithelial cells of hair matrix and ORS, and the volume of DP becomes smaller (Nicu et al., 2020). The DPCs are resistant to apoptosis due to their expression of the anti-apoptotic protein BCL-2 (Nan et al., 2020). During catagen, the degeneration of hair follicles is highly regulated, and a large number of keratinocytes in hair follicles begin to undergo programmed death (Bak et al., 2020). At this stage, melanin production in hair follicles stops and melanin cells in some hair follicles also begin to undergo apoptosis (Bejaoui et al., 2020). By the end of the catagen stage, the hair follicles have atrophied and the DPCs have begun to condense and move upward to the lower part of the bulge area. If the DPCs of a follicle fails to move to a position underneath the bulge area during catagen, the hair follicle no longer undergoes cyclic growth, and this ultimately results in hair loss, which has been confirmed in humans and mice with hair loss gene mutations (Choi et al., 2021; Zhang Y. et al., 2021). The time for humans to first enter catagen occurs in the uterus, while mice enter catagen about 17 days after birth (Paus and Foitzik, 2004).

### 4.3 Telogen

After the catagen stage, the hair follicle enters telogen, when the biological activity of the hair follicle is the weakest and the HS falls off. However, expression and activity of the relevant regulatory factors in the hair follicle governing its cyclical growth will be significantly enhanced to prepare for the beginning of the next anagen. During telogen, the DPCs migrate to the lower part of the bulge, so that the DPCs can interact directly with the stem cells in the bulge. DPCs are essential for the activation of stem cells and the initiation of new hair cycles. After activation, HFSCs proliferate and the number of HFSCs reaches a critical value, the next anagen stage of hair follicles begins (Kim et al., 2022). In the hair cycle of mice, the first telogen is very short, lasting only 1–2 days; the second telogen lasts more than 2 weeks, starting about the 42nd day after birth (Hwang et al., 2021) (Figure 3C).

## 5 The Molecular Regulation of Hair Follicle Morphogenesis and Cycling

A variety of different molecular signaling pathways are involved in governing hair follicle development and cycling, such as the canonical WNT and BMP signaling pathways. Additionaly, miRNAs can also contribute to the regulation of morphogenesis and regeneration of hair follicles. Different signal pathways and factors combine to form a complex molecular regulatory network, the activity of which results in the proper morphogenesis and regeneration of hair follicles.

### 5.1 Signaling Pathways in the Morphogenesis and Cycle of Hair Follicles

Signaling pathways regulate hair follicle morphological development and cycles strictly. And when disturbed, hair follicle-related diseases will develop. WNT, BMP, EDAR, and Sonic hedgehog (Shh) are considered the main pathways involved in regulating follicle morphogenesis, while other pathways are thought to influence morphogenesis as well (Rishikaysh et al., 2014). When the ligand, receptor and signal transduction molecules of these signal pathways are abnormal, the development of animal hair follicles will be affected, leading to changes in hair growth. Genes reported to promote the early morphogenesis of hair follicles include WNT/β-catenin, WNT10b, LEF1, and EDAR as expressed in the epidermis; and WNT/β-catenin, WNT5a, LEF1, and Noggin as expressed in the dermis. Genes believed to inhibit the early morphogenesis of hair follicles include DKK4 and BMP2 as expressed in the epidermis; and DKK1, BMP4, and BMP7 as expressed in the dermis (Albrecht et al., 2021; Huang J. et al., 2021).

#### 5.1.1 WNT Signaling Pathway

The WNT pathway is one of the most important signaling pathways regulating hair follicle morphogenesis and cycle. It is also the earliest known signaling pathway to initiate the induction of hair follicle development by regulating the formation of the basal plate (Zhao et al., 2022). Canonical WNT signaling pathway mainly includes WNT protein, cell surface Frizzled receptor family, Dishevelled (DSH) receptor family protein, β-catenin, and axin/GSK-3/APC complex.

**FIGURE 4:**
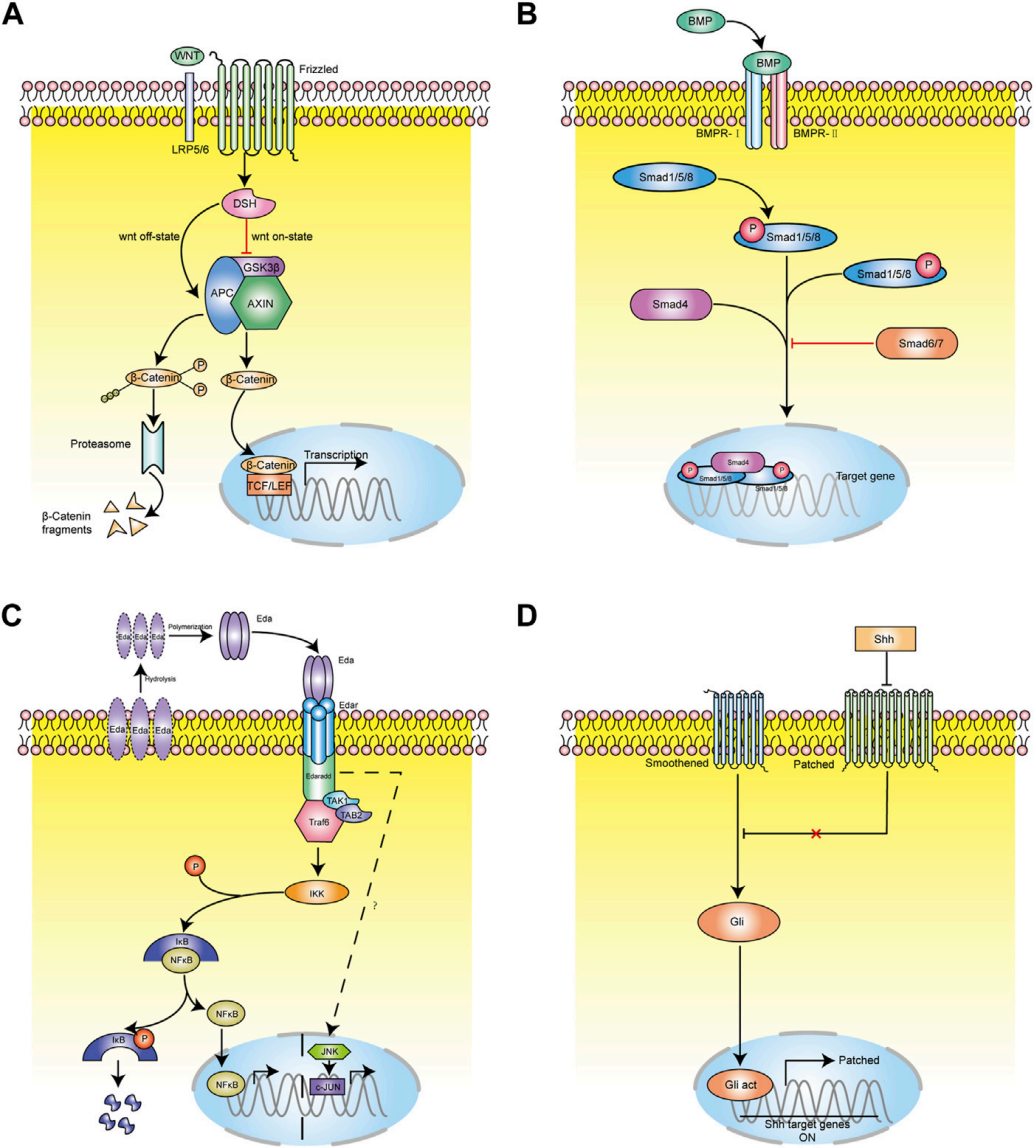
The molecular regulation of hair follicle morphogenesis and cycling. **(A)** WNT signaling path mode diagram. Schematic drawing illustrating canonical WNT signaling pathway mainly includes WNT protein, cell surface Frizzled receptor family, Dishevelled (DSH) receptor family protein, β-catenin, and axin/GSK-3/APC complex. β-catenin plays a key role in the canonical WNT signaling pathway. **(B)** BMP signaling pathway mode diagram. The classical BMP signaling pathway is that ligand BMP binds to phosphorylated serine and threonine receptors and is transported into cytoplasm. In cytoplasm, BMP combines with Smad1/5/8 and phosphorylates the C terminal of Smad1/5/8. The phosphorylated Smad1/5/8 combines with Smad4 and transports to the nucleus. **(C)** EDAR signaling path mode diagram. EDAR signaling pathway is mainly composed of EDA ligand, transmembrane receptor EDAR (including EDAA1 and EDAA2 subtypes), and intracellular binding protein EDARARR. **(D)** SHH signaling path mode diagram. Ptch binds to Smo to inhibit Smo activity. In the presence of Hh, the binding of Ptch1 to Hh protein eliminates the inhibitory effect on Smo, and Smo transmits signals to downstream Gli transcription factors through a complex transduction process.

WNT is a secretory glycoprotein with more than 20 related family members. Its secretion is mediated by wntless (Wls), which is a transmembrane transporter. Although the role of Wls in the induction of hair follicle development is still unclear, it has been found to exist in embryonic epithelium and hair follicles after hair formation (Huang et al., 2012). Studies have found that the WNT family can be divided into primary WNT and secondary WNT. The primary WNT includes WNT3, WNT4, and WNT6. WNT3a is only expressed in bulge and decreased in expression during catagen, and not is expressed at all during telogen (Li et al., 2021; Xing et al., 2018). Guo et al. (2012) found that melanocytes in hair follicles express both WNT3a and β-catenin proteins at the same time. It is speculated that they play an important role in the proliferation, differentiation, and pigment deposition of hair follicle melanocytes. The secondary WNT includes WNT2, WNT7b, WNT10a, and WNT10b. Primary WNT is necessary for the induction of hair follicles, while secondary WNT mainly plays a role in the development of hair follicles (Nicu et al., 2021; Zhang W. et al., 2021). WNT10b mainly plays a role in the mammalian hair follicle cycle and is highly expressed during anagen of the hair follicle, thus promoting epithelial differentiation and early development of hair follicles (Bai et al., 2021). During telogen, overexpression of WNT10b can induce hair follicles to change from refractory phase to inductive phase, thus entering anagen (Hawkshaw et al., 2019; Liu et al., 2021). WNT3a is mainly expressed in root sheath progenitor cells, bulbs, hair bulges, epidermis, melanocytes, and melanin stem cells in hair follicles. Chen et al. (2015) found that the expression of tumor necrosis factor α (TNF- α) increased after hair removal, while TNF- α related peptides could significantly stimulate keratinocytes to express WNT3, WNT10a and WNT10b. TNF- α promotes hair regeneration by activating the NF- κB signaling pathway and finally activating the WNT signaling pathway. β-catenin is an important mediator of the canonical WNT signaling pathway (Figure 4A). It is mainly expressed in ORS, IRS, hair matrix, HS, and other structures of hair follicles. DPCs, hair matrix cells, and ORS cells express β-catenin at high levels (Zhao et al., 2022). β-catenin is the core signal transduction factor in WNT signaling pathway. During hair follicle regeneration, β-catenin expressed in HFSCs in the hair germ and bulge activates the LEF/TCF complex, which further initiates the transcription of downstream target genes c-myc and cyclinD1 involved in cell cycle control and apoptosis, thus promoting the activation, proliferation, and directional differentiation of HFSCs (Lin et al., 2015). WNT signaling protein is mainly expressed during hair follicle anagen, decreased during catagen, and inactivated during telogen.Zhu et al. shows that LncRNA H19 plays a role by directly down-regulating the expression of WNT inhibitors DKK1, Kremen2 and SFRP2, and inducing miR-29a to activate WNT signal, thus forming a new regulatory feedback loop between H19 and miR-29a to maintain hair follicle induction potential. LncRNAH19 maintains the hair follicle induction ability of dermal papilla cells by activating WNT pathway, which may be a target for the treatment of androgenic alopecia (Zhu et al., 2020). Blimp1 is both a target and a mediator of key dermal papilla inductive signaling pathways including transforming growth factor-β and WNT/β-catenin (Telerman et al., 2017).

#### 5.1.2 BMP Signaling Pathway

The BMP pathways is another key signalling related to hair follicle morphogenesis and cycle. Mou et al. (Telerman et al., 2017) found that in skin tissue culture, treatment with BMP led to the formation of hair follicle base plate, while treatment with the BMP antagonist Noggin could increase the density of hair follicle base plate, which further proved the inhibitory effect of BMP on hair growth. Studies have found that the use of Noggin (a BMP antagonist) in mouse skin can significantly shorten the refractory period and promote hair regeneration, so BMP may play a role as an inhibitory signal of hair growth (Plikus et al., 2008). BMP is a family of secreted glycoproteins, belonging to the transforming growth factor (TGF) superfamily, and are multifunctional growth factors. It activates signal transduction by binding with BMP receptors (Figure 4B) (Monsivais et al., 2021). A serine-threonine kinase receptor, which form an active kinase heterotetrameric receptor complex after combination with BMP. This activated kinase complex phosphorylates the C-terminus of Smad1/5/8 (Infarinato et al., 2020), which subsequently binds to Smad4 and is transported to the nucleus. With the cooperation of other transcription factors, the transcription of target genes of Smad1/5/8 is initiated to regulate the proliferation and differentiation of HFSCs (Olsen et al., 2020). Smad6 and Smad7 are inhibitory Smad proteins, which block BMP signal transduction by competing with Smad4 to bind Smad1/5/8, and can also cause ubiquitin degradation of Smad1/5/8 (Chen et al., 2021). The BMP signaling pathway acts on the refractory period and regeneration period in telogen, during which BMP has different signal activity intensity. Moreover, Plikus et al. (2017) found that myofibroblasts can reprogramming to adipocytes during wound healing by the participation of new hair follicles. And this process triggers the BMP signaling pathway, thus further activating the expression of adipocyte transcription factors during development.The BMP pathway has been reported to play a positive role in determining the glandular fate during the induction stage of eccrine sweat gland. additionaly, the eccrine sweat glands were converted to hair follicle-like structures in Bmpr1a conditional knockout mice (Lu et al., 2016).

#### 5.1.3 EDAR Signaling Pathway

EDAR is also one key signalling pathway related to the development and cycle hair follicle. It is mainly composed of EDA ligand, transmembrane receptor EDAR (including EDAA1 and EDAA2 subtypes), and intracellular binding protein EDARARR (Figure 4C) (Wang et al., 2020). EDAR and EDA belong to the TNF superfamily. EDAR contains extracellular ligand binding N-terminal, single transmembrane domain, and intracellular death domain (Schuepbach-Mallepell et al., 2021). Its death domain can specifically bind to the extracellular domain of intracellular binding protein EDARARR, initiate signal transduction, and regulate the transcription of downstream target genes (Wohlfart and Schneider, 2019). In the hair follicle cycle of wild-type mice, the expression of EDA, EDAR, and EDARADD reaches the peak at the end of anagen, decreases from the end of catagen to the middle stage, and was the lowest in telogen (Fessing et al., 2006). At the end of anagen, Eda-A1 was expressed in hair matrix, IRS, and ORS. In the middle stage of catagen, the expression of Eda-A1 decreased sharply and was only expressed in the hair buds of secondary hair follicles. EDAR was expressed in the hair matrix and the IRS at the middle stage of the anagen, but at the end of anagen, the expression of EDAR in the IRS and the ORS increased rapidly. At the beginning of catagen, EDAR was expressed only in the IRS and ORS, but at the end of catagen, EDAR only appeared in the hair buds of secondary hair follicles (Gomez et al., 2013).

#### 5.1.4 Shh Signaling Pathway

Shh is a small secreted glycoprotein that is frequently involved in inducing cell proliferation, cell fate determination, and patterning in a number of developing tissues. Transmembrane proteins Patched (Ptch) and Smoothened (Smo) are two transmembrane proteins components of Shh signaling (Figure 4D) (Morinaga et al., 2021). In the absence of Shh protein, Ptch binds to Smo to inhibit Smo activity. In the presence of Shh, the binding of Ptch1 to Shh protein eliminates the inhibitory effect on Smo, and Smo transmits signals to downstream Gli (glioma-associated oncogene homologue) transcription factors through a complex transduction process, activating Gli and allowing transport of Gli into the nucleus (Sun et al., 2021). After activated Gli enters the nucleus, it initiates the transcriptional expression of downstream Cyclin D1 and N-myc genes (Sigafoos et al., 2021).

Shh participates in the morphogenesis of hair follicles during embryonic development. The DPCs of Shh mutant mice were reduced in number, and these mice lacked normal hair follicles so they could not maintain normal hair morphology (Lim et al., 2018). Dermal expression of Shh is critical for maturation of the DP and maintaining expression of DP-specific genes during morphogenesis (Woo et al., 2012). Shh continues to participate in the regulation of follicle cycling in adults by promoting the transition of hair follicles from telogen to anagen (Gao et al., 2019; Zhang X. et al., 2021). Studies using an anti-Shh monoclonal antibody that disrupts Shh activity show destruction of hair follicles during anagen and subsequent hair loss. This supports the idea that the Shh signaling pathway plays a key role in the hair growth of mice (Choi, 2018; Zhang X. et al., 2021). Shh secreted from perifollicular nerve endings is also important for the maintenance of Gli1^+^Lgr6^+^ stem cells present in the follicle that can contribute to epidermal healing after wound formation (Brownell et al., 2011). The varied effects of Shh in the touch dome to the ligand source, with locally produced Shh acting as a morphogen essential for lineage specification during development and neural Shh regulating postnatal touch dome stem cell maintenance (Xiao et al., 2016).

### 5.2 miRNAs Regulating Hair Follicle Morphogenesis

MiRNA is expressed in the skin and hair follicles of mammals and plays an important role in regulating the development and regeneration of hair follicles (Andl and Botchkareva, 2015; Hochfeld et al., 2017; Horsburgh et al., 2017). Mice lacking dicer enzyme could not form normal miRNA, resulting in the formation of hair bud-like cysts (Yi et al., 2006) in the epidermis. The miR-24 can affect the differentiation of mouse HFSCs by inhibiting Tcf-3 during the hair follicle anagen. The hair of mice with ectopic expression of miR-24 becomes thinner and these mice develop serious defects in hair follicle development (Amelio et al., 2013). The expression of miR-22 during catagen and telogen is higher than that in anagen. miR-22 regulates hair follicle cyclical changes and affects the formation of IRS and HS by inhibiting the expression of transcription factors DLX3, FOXN1, and HOXC13 (Cai et al., 2020; Yuan et al., 2021). MiR-125b can inhibit the expression of target genes Blimp1 and Vdr, resulting in the inhibition of the differentiation of HFSCs and promoting stem cell renewal (Zhang et al., 2011). BMP4 has an inhibitory effect on hair follicle development, and the expression of miR-21 can weaken this effect (Xiong et al., 2022).

## 6 Perspectives

The morphogenesis and grow cycling of hair follicles involve many cells and molecules. These signaling molecules are not independent, and various studies have shown that they are formed into complex regulatory network. Therefore, the search for key signaling molecules that control these processes has become a major focus of hair research. Currently, treatment with certain medications, over-expression or inhibition of endogenous genes and increasing the secretion of extracellular vesicles (EVs) are the main strategies to promote hair growth, which involve a variety of signaling pathways and molecules. All these findings provide new ideas for the clinical treatment of hair follicle related diseases such as alopecia areata. Despite significant advances in this field, what are the key activators that promote the transformation of hair follicle telogen and anagen stages through signaling pathways and what is the molecules mechanism of hair growth promotion through HFSCs and dermal stem cells are still unclear. Moreover, although the transplantation of potential cell mixtures, hair follicle organoid construction *in vitro*, reprogramming induction and the establishment of a drug delivery system do contribute to the formation of hair follicles, the construction of functional hair follicles with normal cycling activity is still a great challenge for the hair research field. Therefore, further studies are still needed to on the activation and maintenance of hair cycling in the next phase.
